# Deep mutational scanning of the plasminogen activator inhibitor-1 functional landscape

**DOI:** 10.1038/s41598-021-97871-7

**Published:** 2021-09-22

**Authors:** Zachary M. Huttinger, Laura M. Haynes, Andrew Yee, Colin A. Kretz, Matthew L. Holding, David R. Siemieniak, Daniel A. Lawrence, David Ginsburg

**Affiliations:** 1 Life Sciences Institute, University of Michigan, Ann Arbor, MI USA; 2 Cellular and Molecular Biology Program, University of Michigan Medical School, Ann Arbor, MI USA; 3 Department of Pediatrics, Baylor College of Medicine, Houston, TX USA; 4 Department of Medicine, McMaster University and the Thrombosis and Atherosclerosis Research Institute, Hamilton, ON Canada; 5 Howard Hughes Medical Institute, Ann Arbor, MI, USA; 6 Department of Internal Medicine, University of Michigan Medical School, Ann Arbor, MI USA; 7 Department of Molecular and Integrative Physiology, University of Michigan Medical School, Ann Arbor, MI USA; 8 Departments of Human Genetics and Pediatrics, University of Michigan, Ann Arbor, MI USA; 9 Present Address: Department of Otolaryngology, The Ohio State University College of Medicine, Columbus, OH USA

**Keywords:** Proteases, High-throughput screening, Next-generation sequencing, Thrombosis

## Abstract

The serine protease inhibitor (SERPIN) plasminogen activator inhibitor-1 (PAI-1) is a key regulator of the fibrinolytic system, inhibiting the serine proteases tissue- and urokinase-type plasminogen activator (tPA and uPA, respectively). Missense variants render PAI-1 non-functional through misfolding, leading to its turnover as a protease substrate, or to a more rapid transition to the latent/inactive state. Deep mutational scanning was performed to evaluate the impact of amino acid sequence variation on PAI-1 inhibition of uPA using an M13 filamentous phage display system. Error prone PCR was used to construct a mutagenized PAI-1 library encompassing ~ 70% of potential single amino acid substitutions. The relative effects of 27% of all possible missense variants on PAI-1 inhibition of uPA were determined using high-throughput DNA sequencing. 826 missense variants demonstrated conserved inhibitory activity while 1137 resulted in loss of PAI-1 inhibitory function. The least evolutionarily conserved regions of PAI-1 were also identified as being the most tolerant of missense mutations. The results of this screen confirm previous low-throughput mutational studies, including those of the reactive center loop. These data provide a powerful resource for explaining structure–function relationships for PAI-1 and for the interpretation of human genomic sequence variants.

## Introduction

**Figure 1:**
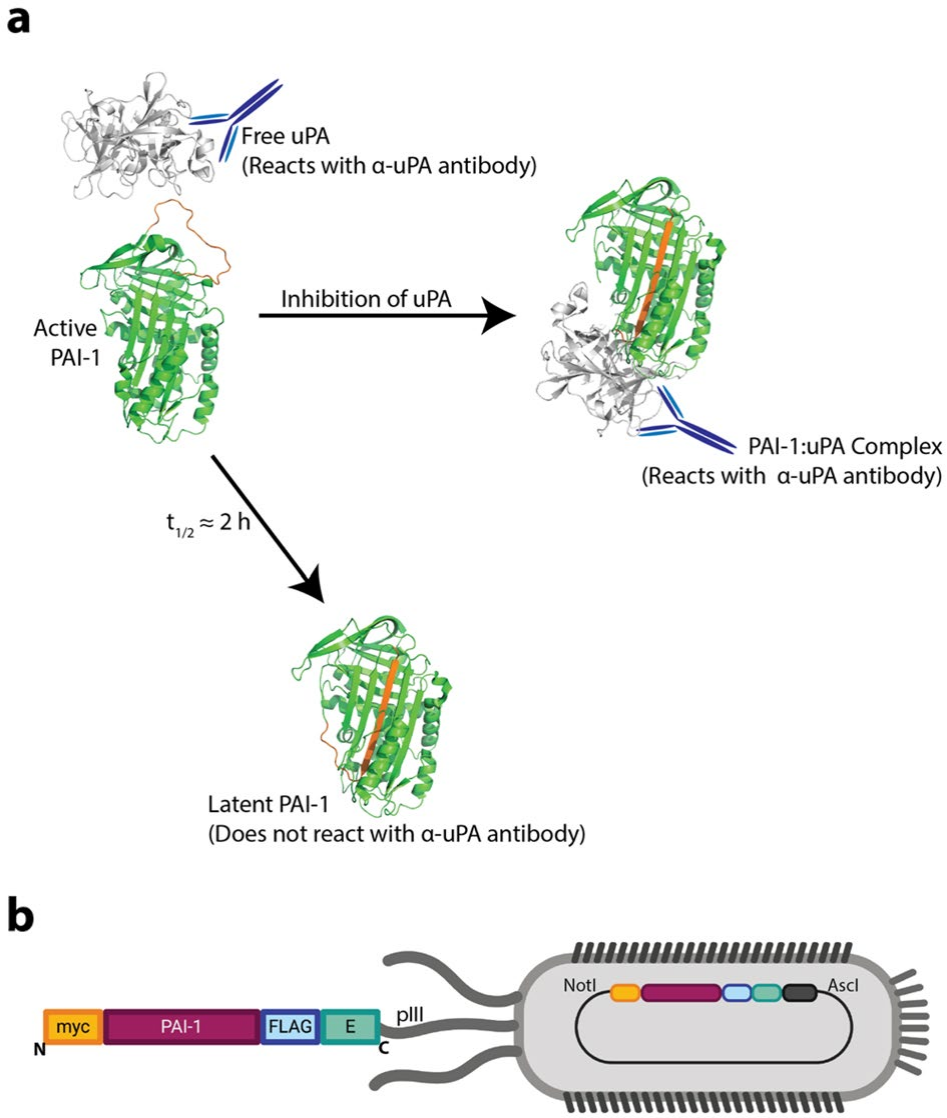
PAI-1 displayed as a fusion protein on the surface of filamentous phage is an inhibitor of uPA. (**a**) Active PAI-1 (PDB:3PB1) reacts with free uPA (PDB:1W0Z) to form a covalent uPA:PAI-1 complex^59,60^. Both free and complexed uPA can be immunoprecipitated using an anti-uPA antibody. Alternatively, PAI-1 can spontaneously relax from its active, metastable state to a low-energy yet chemically inert latent conformation (PDB: 1DVN)^61^. PAI-1’s reactive center loop is highlighted in orange. (**b**) Schematic of phPAI-1 displayed as a fusion to the pIII coat protein of M13 filamentous phage, with the N-terminal myc- and C-terminal FLAG- and E-tags highlighted. This figure was generated using Adobe Illustrator 2021 version 25.2.3 (**a**,**b**), PyMOL version 2.5.0 (**a**), and BioRender.com (**b**).

Plasminogen activator inhibitor-1 (PAI-1, *SERPINE1*) is a member of the serine protease inhibitor (SERPIN) protein superfamily^1^. SERPINSs function as irreversible inhibitors that covalently bind the active site of their target proteases^2^. All inhibitory SERPINs share a common mechanism wherein a flexible reactive center loop (RCL) extends outside the central structure of the molecule serving as a cleavable peptide “bait” for the target protease (Fig. 1a)^3^. The SERPIN and protease form an acyl intermediate when the active site serine acts as a donor nucleophile to bind to the carbonyl carbon of the P1 residue within the RCL^4,5^. However, the RCL rapidly inserts into central β-sheet A before the hydrolysis reaction is completed, carrying the tethered protease to the opposite side of the molecule in a pole-to-pole transition^6^. This structural transformation stabilizes the protease-SERPIN complex and renders both the SERPIN and the serine protease no longer active^2,7,8^. If the rate of RCL insertion is slower than the resolution of the acyl intermediate, complete proteolysis of the scissile bond occurs, with the SERPIN then functioning as a substrate instead of an inhibitor^9^. Either of these mechanisms is possible when a SERPIN interacts with a given protease. The specificity of a SERPIN for its target protease is in part driven by the probability that the inhibitory pathway dominates over the substrate pathway^10^. This balance presents a unique challenge when engineering SERPIN variants that inhibit non-canonical target proteases^11,12^.

PAI-1 is the principal inhibitor of the serine proteases tissue-type and urokinase-type plasminogen activators (tPA and uPA, respectively)^13,14^. tPA and uPA activate the zymogen plasminogen to plasmin^15^, the enzyme responsible for the proteolysis of fibrin—the structural backbone of blood clots^16^. Human deficiency of PAI-1 results in excessive plasmin generation and a mild to moderate bleeding diathesis^17–21^. Likewise, PAI-1 deficient mice are viable, exhibiting a moderate increase in fibrinolytic activity^22^. In addition to its critical role in regulating hemostasis, PAI-1 has been implicated in a number of other processes, including a link to longevity^23^ and numerous other pathogenic processes^24^. Given the wide-ranging functions of PAI-1, understanding the effects of potentially damaging mutations in PAI-1 may have clinical implications beyond the canonical role of PAI-1 in regulating fibrinolysis.

The present work couples phage display with high-throughput DNA sequencing (HTS), to measure the effects of multiple missense mutations on PAI-1’s uPA inhibitory function in a massively parallel fashion^25^. The overall goal is to map the mutational landscape of PAI-1 with respect to mutations that render PAI-1 no longer functional to (1) better understand the natural evolution of PAI-1 with respect to the amino acid space that can be occupied at any given residue, but also (2) to use this high resolution map of PAI-1 as a basis for engineering novel SERPINs. SERPINs, including PAI-1, have been previously engineered to inhibit proteases other than their canonical targets^26,27^ demonstrating the potential to develop novel therapeutics for the treatment of a variety of disorders including hemophilia and alpha-1-antitrypsin deficiency. PAI-1 is a particularly attractive choice as a SERPIN scaffold as it lacks native cysteine residues and remains functional in the absence of glycosylation, facilitating large-scale production of functionally active, recombinant PAI-1 in bacterial systems^28,29^.

## Results and discussion

### Characterization of PAI-1 fusion protein

**Figure 2:**
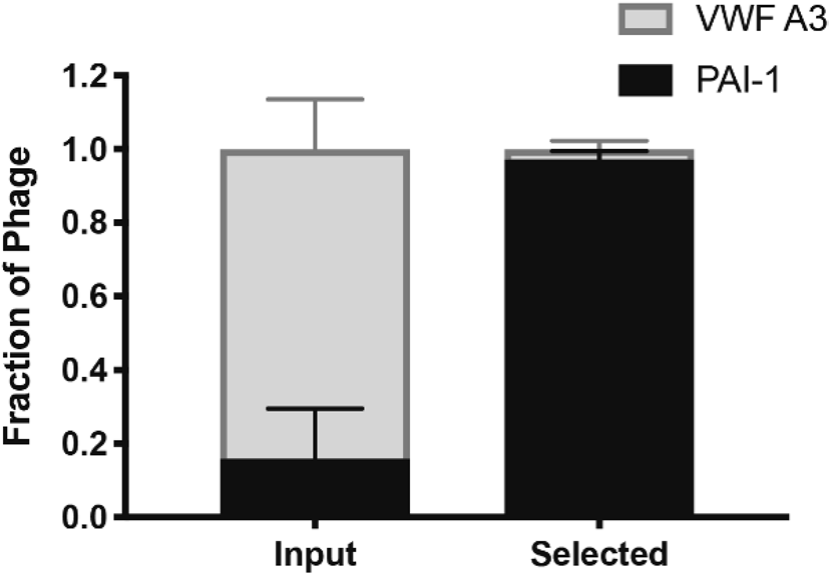
Immunoprecipitation is specific for phPAI-1:uPA complexes. Nine parts phVWF-A3 and one part phPAI-1 (9:1) were combined (*input*, n = 3), incubated with uPA (1.7 nM) for 30 min at 37 °C, and selected by immunoprecipitation with an anti-uPA antibody (*selected*, n = 3). For each replica, 24 colonies were genotyped by PCR using primers common to both the phVWF-A3 (677 bp) and phPAI-1 inserts (1271 bp) followed by analysis on a 1% agarose gel. This figure was generated using GraphPad Prism version 9.0.2.

Selection of phage displayed PAI-1 (phPAI-1) in the presence of a ninefold molar excess of a negative control, phage displaying the A3 domain of von Willebrand Factor (VWF, a protein fragment not known to interact with uPA, phVWF-A3), for complex formation with uPA (Fig. 2) resulted in at least a five-fold enrichment in phPAI-1 relative to phVWF-A3, indicating that the immunoprecipitation is specific for uPA and uPA:PAI-1 complexes. Given the rigorous washing of the immunoprecipitated complex, this enhancement further suggests that PAI-1 expressed as a pIII fusion protein on the phage surface retains its inhibitory activity^30–32^.

### Characterization of the mutant library

**Figure 3:**
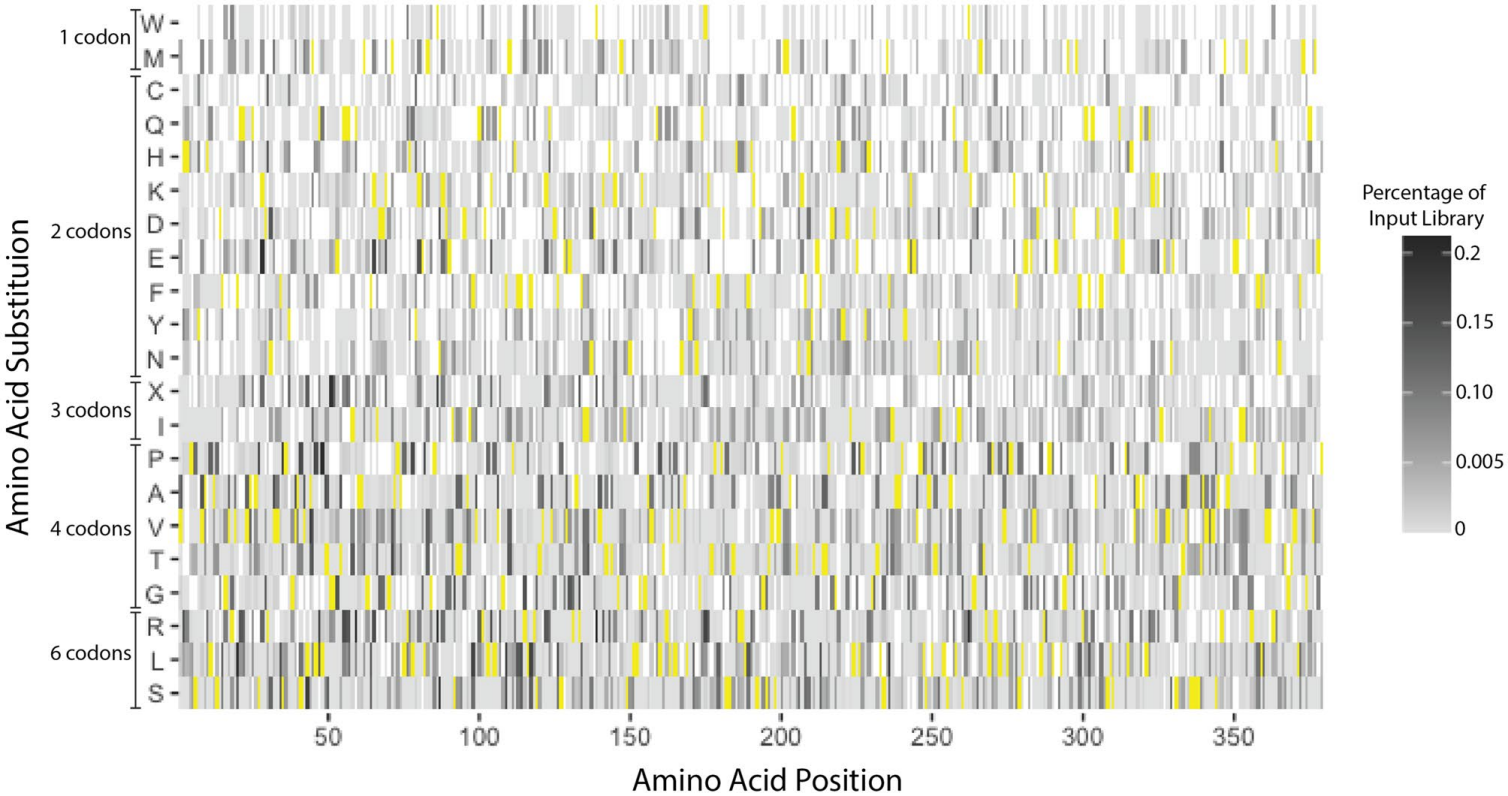
phPAI-1 mutant library generated by error prone PCR includes more than two-thirds of all possible missense mutations. The mutational library contains 71% of all possible missense and nonsense mutations with 27% of all missense variants present with sufficient depth (base mean score > 10, p_adj_ < 0.05) to accurately determine the effects of the mutation on PAI-1 function. The primary amino acid position within PAI-1 is indicated along the x-axis and single amino acid substitutions are listed along the y-axis. WT amino acid residues are indicated in yellow, while missense and nonsense (X) mutations not present in the input library are shown in white. Variants present within the library are shown in grey (see scale) as a percentage of the input library represented by that variant. This figure was generated using Adobe Illustrator 2021 version 25.2.3 and the ggplot2 package version 3.3.3 for RStudio version 1.4.1106.

The phPAI-1 mutant library exhibits a depth of 8.04 × 10^6^ independent clones with an average of 4.2 ± 1.8 amino acid substitutions/molecule as determined by Sanger sequencing of 13 randomly selected phage clones. HTS demonstrated that 5117 of the possible 7201 missense variants (71%) are present in the mutant phPAI-1 library, along with at least 1 nonsense mutation at 269 of the 379 PAI-1 amino acid positions (71%). The frequency of DNA sequencing reads for individual amino acid substitutions within the starting library ranged over > 10^4^-fold. As expected, the frequency of specific amino acid substitutions also varied based on the genetic code, with, for example, reduced representation of Met and Trp substitutions (both encoded by only a single codon), compared to Arg, Leu, and Ser substitutions (each encoded by six codons) (Fig. 3).

**Figure 4:**
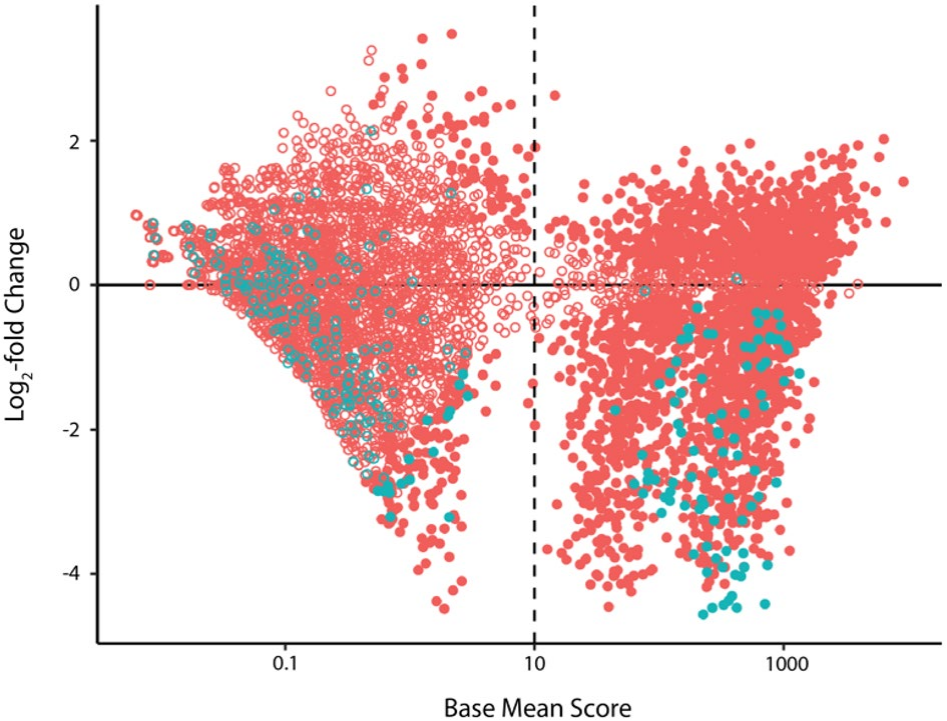
phPAI-1 uPA-selected libraries are enriched in variants that retain their inhibitory function and depleted of those without inhibitory activity. An MA plot^33^ of base mean score (average of counts in the input and selected libraries) vs. log_2_-fold change is shown, with missense mutations in pink and nonsense variants in blue. Variants with p_adj_ < 0.05 are shown as closed circles, while those that do not meet this significance threshold are shown as open circles. A base mean score greater than 10 was also set as a threshold for determining significance. This figure was generated using Adobe Illustrator 2021 version 25.2.3 and the ggplot2 package version 3.3.3 for RStudio version 1.4.1106.

To limit the proportion of phPAI-1 variants transitioning to the non-reactive latent conformation, all reactions with uPA were performed immediately following phage production. To limit false positives within the dataset, only those variants with a base mean score (average of the normalized counts in the input and selected libraries corrected for sequencing depth as defined by Love et al.^33^). greater than 10 and an adjusted p value (p_adj_) < 0.05 were included in further analyses (Fig. 4). Based on these criteria, 1963 (38%) of the 5117 missense variants present in the starting library could be scored for uPA reactivity or lack thereof. Although not a complete profile of all mutational space, these data represent a marked expansion of the mutational space that has been explored in previous reports^34^. Furthermore, the use of HTS facilitates accurate assessment for both gain- and loss-of-function mutations after only a single round of panning^31,35^.

### Massively parallel assessment of variant impact on uPA inhibitory function

**Figure 5:**
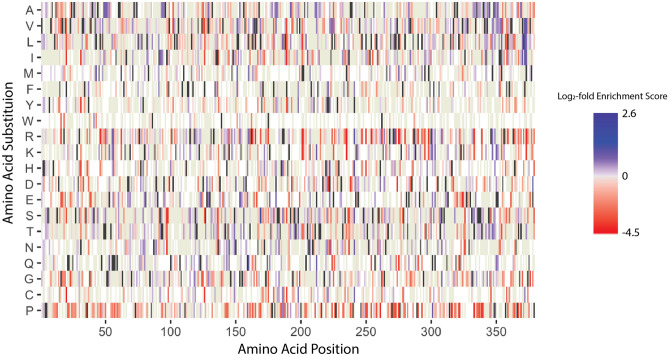
phPAI-1 mutational libraries contain missense variants that result in loss of function. Amino acid position is indicated on the x-axis, while amino acid substitutions are indicated on the y-axis. Loss of function missense variants, as well as those with a reduced capacity to inhibit uPA, are shown in red with shading as a function of their log_2_-fold score, while variants in blue retain PAI-1 inhibitory function. The intensity of shading scales with the degree of enrichment or depletion. WT amino acid residues are shown in black, while beige indicates missense mutations that were present in the mutational library but did not meet significance thresholds. White indicates amino acid substitutions that were not present in the mutational library. This figure was generated using Adobe Illustrator 2021 version 25.2.3 and the ggplot2 package version 3.3.3 for RStudio version 1.4.1106.

Following selection with uPA, 826 PAI-1 missense variants retained the ability to form a complex with uPA, with a range of enrichment scores likely representing varying degrees of inhibitory activity towards uPA (Fig. 5). Similarly, depleted variants (log_2_-fold enrichment score ≤ 0, n = 1137) are broadly classified here as loss-of-function, likely including variants that retain a low level of inhibitory activity towards uPA—again, reflecting that this approach enables the mapping of functional variability with respect to both gain and loss of function (Fig. 5). Missense variants were enriched or depleted up to 6- or 23-fold, respectively and different amino acid substitutions at the same position may exhibit opposite effects. For comparison, consider two mutations at Ile^91^, I91L and I91N, each representing approximately 0.0005% of the of the input library (Fig. 3). Following selection with uPA, I91L was enriched three-fold, consistent with previous reports that this mutation not only does not ablate PAI-1’s inhibitory function, but also extends its functional half-life^31^. In contrast, I91N was depleted three-fold—demonstrating that while the I91L mutation is well tolerated, I91N results in loss of function with respect to uPA inhibition. Of note, the selection method employed here (complex formation with uPA) does not distinguish between the three potential mechanisms for loss-of-function: PAI-1 misfolding, accelerated transition to the inactive latent state, and/or serving as a substrate for uPA. All three of these loss-of-function phenotypes would result in the inability of PAI-1 to form a covalent complex with uPA, and thus would be lost to selection for uPA binding.

### Implications for structure–function relationships

**Figure 6:**
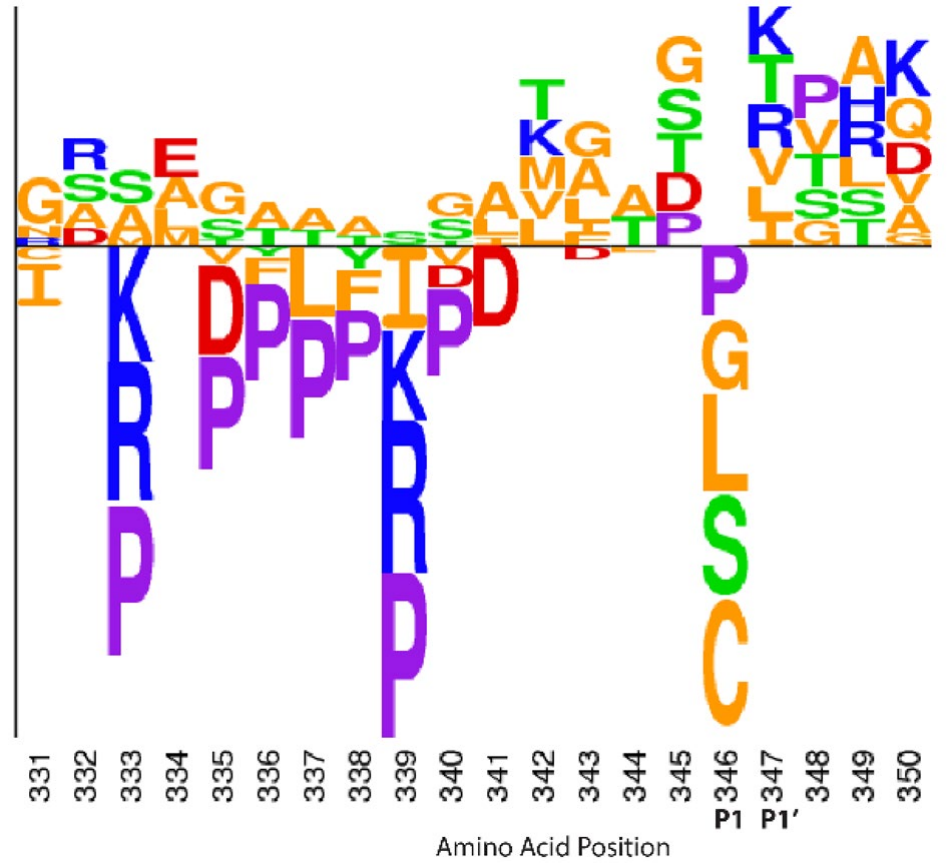
Deep mutational scanning of PAI-1 provides insight into the mutational landscape of PAI-1’s RCL. Relative log_2_-fold change scores of missense mutations in PAI-1’s RCL (amino acids 331–350) following selection by ability to inhibit uPA^39^. Amino acids below the x-axis indicate depletion (log_2_-fold change < 0), while those above the x-axis indicate enrichment (log_2_-fold change > 0). Letter height corresponds to the relative log_2_-fold change. Residues are color coded by properties: acidic residues in red, basic residues in blue, polar amino acids in green, non-polar amino acids in orange, and Pro in purple^39^. The P1 and P1′ positions (Met^346^ and Arg^347^ in WT) are also indicated. This figure was generated using Adobe Illustrator 2021 version 25.2.3 and Seq2Logo 2.0 (http://www.cbs.dtu.dk/biotools/Seq2Logo/).

The results of our PAI-1 functional screen can be used to assess specific regions within PAI-1 without the additional construction of a targeted libraries. This point is highlighted by analysis of the RCL (residues 331–350) as illustrated in Fig. 6, although a similar approach could similarly be applied to other regions of interest, In the RCL, the observed enrichment and depletion at the P1 and P1′ positions (residues 346 and 347) are consistent with our understanding of PAI-1 biology. The P1 position has been shown to be a key determinant of SERPIN target protease specificity^36–40^, with PAI-1 inhibitory activity toward uPA requiring either a P1 Lys or the WT Arg residue^41^ Consistent with these previous reports, no missense mutations were tolerated at P1 in our screen (of note, lysine at this position is absent from our library), with several substitutions significantly depleted (Figs. 3, 6). Consistent with the previously reported tolerance of the P1′ position for most amino acid substitutions^41^, our screen identified no loss-of-function PAI-1 variants at this position (Fig. 6).

At the N-terminus of the RCL, enriched or tolerated substitutions observed in our data generally consist of small aliphatic and polar amino acids. For PAI-1 to retain its inhibitory function, this region of the RCL must be able to insert into β-sheet A^9^. These small amino acids allow the RCL to undergo the dramatic conformational changes that are required for this insertion. Consistent with this model, substitutions with bulky and/or charged side chains (Lys, Arg, Pro, Asp, Phe) were the most depleted residues for those N-terminal RCL positions whose side chains become oriented into the core beta sheet upon insertion^42–45^. In contrast, residues C-terminal to the scissile bond (P2′–P4′) are more tolerant of mutations than those at the N terminus of the RCL, as the former region does not insert into the central β-sheet^46^. Finally, the flexibility of the RCL is also important for dictating PAI-1’s inhibitory behavior, and our data are concordant with a previous proline-scanning mutagenesis screen^42^. Proline residues in the RCL would also be incompatible with RCL insertion into β-sheet A, which transforms it from a largely parallel β-sheet to a more stable anti-parallel β-sheet.

### Correlation with predictive algorithms and human genome sequence variant data

A number of algorithms have been developed to predict the impact of single amino acid substitutions on protein function based on evolutionary conservation and/or amino acid type^47^. We compared our high throughput screening data with predictions from two commonly used algorithms, SIFT^48^ and PolyPhen-2^49^. SIFT predicts the effects of amino acid substitutions by comparison to homologous sequences, and PolyPhen-2 uses both sequence conservation and structural homology to predict the effects of amino acid substitutions on protein function.

The SIFT algorithm prediction was concordant for 745 of the 1137 (66%) amino acid substitutions scored as “loss of function” in our screen and for 538 of the 826 (65%) scored as neutral. This level of concordance is similar to that previously reported for known deleterious human genetic mutations in other genes^48^. PolyPhen-2 exhibited concordance with our data for 994 of 1137 (87%) “loss of function” substitutions, but only 454 of the 836 (54%) neutral PAI-1 amino acid substitutions^49^. Overall, while these algorithms are a valuable resource for predicting protein functionality, they are unable to correctly assign all missense variants—emphasizing the need for deep mutational scanning of multiple different types/families of proteins.

Additionally, available human genomic sequence information provides support for the potential value of our data in interpreting the significance of human genetic variation identified by future clinical sequencing. The gnomAD database^50^ catalogs human amino acid sequence variant information from ~ 140,000 human exomic/genomic sequences, including 202 variants scored in our mutation screening analysis. Of these 202 variants, 92 were classified by our data as “loss of function”, significantly less than expected by chance (p = 2 × 10^–4^ SI Table 3), consistent with negative (purifying) selective pressure in the human population to maintain PAI-1 activity.

### Evolutionary conservation of PAI-1 is consistent with mutational tolerance

**Figure 7:**
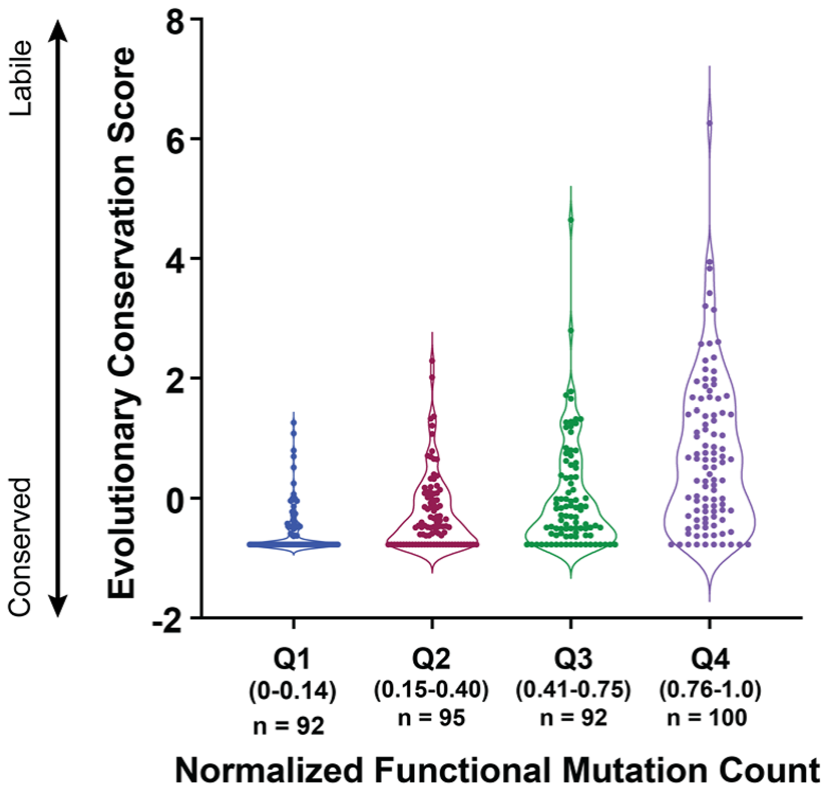
Site susceptibility to accepting missense mutations correlates with evolutionary conservation. The normalized functional mutation count (defined as the number of enriched/functional mutations divided by the total number of mutations scored in our screen at each position) is shown as quartiles along the x-axis with the range and number of positions in each quartile indicated. The fourth quartile (Q4, purple) corresponds to those positions at which the most introduced missense mutations are tolerated, while the first quartile (Q1, blue) corresponds to those positions where the least introduced mutations were tolerated. In comparison the ConSurf evolutionary conservation score is shown on the y-axis and ranges from − 0.77 to 6.3, with larger scores corresponding to sites that are more evolutionarily labile and lower scores corresponding to positions that are more conserved. ANOVA analysis shows that the degree of tolerance for missense mutations predicts evolutionary conservation in extant mammalian species’ PAI-1 (F_3.375_ = 24.5, p < 0.0001 R^2^ = 0.23). This figure was generated using Adobe Illustrator 2021 version 25.2.3 and GraphPad Prism version 9.0.2.

To determine whether the distribution of functional missense mutations detected in this screen reflected the evolutionary constraints of individual PAI-1 amino acid positions, the evolved variation in natural sequences was leveraged. PAI-1 sequences of 84 extant mammalian species were present in the cleaned alignment. Significant differences in evolutionary conservation of sites in these alignments were observed among positions manifesting varying numbers of functional mutants in our mutational scanning data (ANOVA *F*_3375_ = 24.5, *p* < 0.0001, *R*^2^ = 0.23, Fig. 7). The overall trend was toward increasing evolutionary lability (less conservation) in the positions that accepted more functional mutants in our human PAI-1 constructs. For example, position 346 that defines the P1 site is among the most evolutionary conserved residues (evolutionary conservation score = − 0.774) and consequently is also found in the first quadrant of the normalized functional scores (Fig. 7). Natural exploration of sequence space through evolutionary time in PAI-1 therefore provides a partial guide to mutational tolerance that complements deep mutational scanning, which can explore mutational space that is hitherto *unseen* in nature. Overall, these results suggest that there is a limited mutational space that is consistent with PAI-1 functionality as a specific uPA inhibitor, and that altering the specificity of PAI-1 for novel serine proteases will likely require expansion into as yet unexplored regions of PAI-1’s mutational landscape.

## Conclusions

Deep mutational scanning has been applied to a number of proteins to analyze function, binding interactions, cellular protein abundance, cell growth/viability, and protein stability^25,28,29,51,52^. In the present study, we have adapted this approach to construct a detailed map for the mutational landscape of PAI-1 with respect to the gain or loss of its capacity to inhibit uPA. We anticipate that the mutational landscape of PAI-1 for other serine proteases, including its other canonical substrate, tPA, would likely demonstrate significant differences^53^, enabling engineering of PAI-1-like SERPINs with novel inhibitory profiles.

Previous PAI-1 mutational studies^34^ have been restricted to limited segments of PAI-1^41,53^, or selected for a few variants with a unique functional impact, such as extended functional stability^31,35^. The error prone PCR approach used here to generate the phPAI-1 mutant library offers speed and ease of application with broad coverage of a significant subset of potential single amino acid substitutions. However, variant coverage is incomplete (Fig. 3), providing significant loss of function data for only a subset of mutation space (Fig. 5). Future advances in molecular approaches and machine learning algorithms will facilitate a comprehensive map of the mutation landscape for PAI-1 and numerous other proteins^54^.

Broadly, the work presented herein demonstrates how deep mutational scanning complements predictive algorithms of protein function and patterns observed in natural evolutionary processes. Furthermore, the data reported in this study provide a valuable resource for the interpretation of sequence variants in PAI-1 and other genes identified by the expanding clinical application of human whole genome sequencing.

## Methods

### Construction of a phage display library expressing PAI-1 fusion proteins

For display of PAI-1 on the M13 filamentous bacteriophage (phPAI-1, Fig. 1B), human SERPINE1 cDNA including a N-terminal myc tag and Gly–Gly–Gly–Ser linker was cloned between the AscI and NotI restriction sites of pAY-FE (Genbank #MW464120)^55,56^. The resulting construct encodes a phage-displayed PAI-1 protein N-terminally fused to a myc tag and C-terminally fused with FLAG and E tags (Fig. 1). The PAI-1 fusion protein was randomly mutagenized using the GeneMorph II Random Mutagenesis Kit (Agilent Technologies, Santa Clara, CA, USA). Primers used for PCR mutagenesis (SI Table 1) maintained the AscI and NotI restriction sites for ligation of the restriction digested insert into pAY-FE. Following ligation, the library was transformed into electrocompetent XL-1 Blue MRF’ *E. coli* as per manufacturer’s instructions. The depth of the library was determined by quantifying the number of ampicillin resistant colonies. Mutation frequency was estimated by Sanger sequencing of the SERPINE1 inserts from randomly selected individual colonies (n = 13).

### Phage production and purification

Phage were prepared as previously reported^57^. Briefly, *E. coli* harboring pAY-FE PAI-1 were grown in LB Broth supplemented with 2% glucose and ampicillin (100 μg/mL) at 37 °C and during mid-log phase (OD_600_ 0.3–0.4) were infected with M13KO7 helper phage at a multiplicity of infection of ~ 100, followed by growth for an additional 1 h at 37 °C. Cells were pelleted by centrifugation (4250×*g* for 10 m at 4 °C), resuspended in 2xYT media (16 g/L tryptone, 10 g/L yeast extract, 5 g/L NaCl) supplemented with ampicillin (100 μg/mL), kanamycin (30 μg/mL) and IPTG (0.4 mM) to induce expression of the PAI-1 fusion protein, and grown for 2 h at 37 °C^55^. All subsequent phage preparation steps were carried out at 4 °C to minimize PAI-1 transition to latency (SI Fig. 1). Phage were precipitated with polyethylene glycol-8000 (2.5% w/v) and NaCl (0.5 M) for up to 16 h followed by centrifugation (20,000×*g* for 20 min at 4 °C). The precipitated phage pellet was resuspended in 50 mM Tris containing 150 mM NaCl (pH 7.4; TBS). Phage titer was determined by transducing naïve XL-1 Blue MRF’ *E. coli* grown to mid-log phase for 1 h at 37 °C and plating on LB-agar supplemented with ampicillin (100 μg/mL) and 2% glucose.

### Selection of uPA-bound phPAI-1

phVWF-A3, in which the VWF A3 domain (Ser1681-Cys1872) was PCR amplified (SI Table 1) and cloned into the pAY-FE vector between the AscI and NotI restriction sites (generating pAY-FE-VWF-A3), were used as a negative control for uPA binding. phPAI-1 were diluted 9:1 with phVWF-A3 and then incubated with uPA (1.7 nM) for 30 min at 37 °C. Residual protease activity was inhibited by incubating the reaction mixture with 1X EDTA-free protease inhibitor cocktail for 10 min at 37 °C. uPA (free and complexed) was immunoprecipitated using magnetic protein G beads (15 μL), which were previously coupled to a polyclonal anti-uPA antibody (17 nM). Beads were washed four times with TBS containing 5% BSA (1 mL), resuspended in Tris (20 mM) pH 8.0 containing 50 mM NaCl, 2 mM CaCl_2_, and 5% BSA, and eluted by digestion with enteropeptidase (16 U, New England BioLabs, Ipswich, MA, USA) for 16 h at 4 °C. The eluted phage pool was used to infect naive XL-1 Blue MRF’ *E. coli*. Eluted phage titers were quantified by transduction of XL-1 Blue MRF’ cells as described above. To determine the composition of the phage pools before and after selection, single colonies of ampicillin resistant bacteria were selected, and their DNA amplified by PCR using primers annealing outside the insertion site, to a region common to both pAY-FE:PAI-1 and pAY-FE:VWF-A3 (SI Table 1) with three replicates of n = 24 colonies in each.

### High-throughput sequencing (HTS)

Twelve overlapping amplicons (150 bp) were PCR amplified from pAY-FE PAI-1 (SI Table 1) with overlapping regions only analyzed on one amplicon (SI Table 2). PCR amplicon products were gel purified, pooled (100 ng DNA divided equally between 12 amplicons), dA-tailed (NEBNext Ultra End Repair/dA-tail, New England Biolabs), and ligated to NextFlex barcodes (Bioo Scientific, Austin, TX) with NEBNext Ultra Ligation (New England Biolabs). Ligated products were purified with AmPure beads (Beckman Coulter, Indianapolis, IN, USA) according to the manufacturer’s directions. HTS was performed as previously described^29^ using the Illumina HiSeq2500 or HiSeqX platforms (Illumina, San Diego, CA, USA) at the University of Michigan DNA Sequencing Core (Ann Arbor, MI, USA) or MedGenome, Inc (Foster City, CA, USA). HTS data were analyzed using DESeq2^33^ analyzing mutations at each position independent of other mutations within a given amplicon.

### Comparison of variant selection results to publicly available datasets

Results of the DESeq2 analysis of input versus selected were compared to the output from the Sorting Intolerant from Tolerant (SIFT) algorithm that predicts the effect of an amino acid substitution on protein function by multiple sequence alignments of related proteins (PAI-1 from *S. scrofa, B. taurus, M. vison, R. norvegicus,* and *M. musculus;* glia-derived nexin from *H. sapiens, M. musculus, and R. norvegicus;* neuroserpin from *H. sapiens, G. gallus, R. norvegicus, and M. musculus*)^58^. To compare SIFT results to our data, tolerated mutations were defined as those that were able to inhibit uPA (log_2_-fold change > 0) at 0 h, and noninhibitors (log_2_-fold < 0) at 0 h were defined as not tolerated. Finally, a χ^2^ test (SI Table 3) was used to determine if the variants identified as loss-of-function by our high-throughput screen were significantly underrepresented in the gnomAD database^50^ by comparing the expected frequency of variants identified in our screen that were present in gnomAD versus those that were not present.

### Evolutionary variability of PAI-1

A protein alignment of PAI-1 orthologs from 94 mammal species was constructed using the Comparative Genomics tools of the Ensembl webserver (www.ensembl.org; release 104) to search for orthologs to the human serpine1 gene (ENSG00000106366). We trimmed the alignment to include only the 379 positions contained in human PAI-1, and removed sequences containing more than 5 percent gaps after trimming. We then used ConSurf (https://consurf.tau.ac.il/) to calculate evolutionary conservation scores for each position in the protein, where higher ConSurf scores indicated more evolutionarily variable positions. To relate the functional susceptibility of each amino acid position to substitutions in our library to the same position’s evolutionary conservation score, a normalized functional mutation score was determined. This score was equivalent to the number of significantly enriched or functional missense mutations at a given position divided by the total number missense mutations with sufficient depth to be analyzed in our library at the same position. The normalized functional mutation scores were further divided into quartiles and used for an analysis of variance (ANOVA) to test the normalized functional mutation score predicted the degree of evolutionary conservation at the same position.

## Supplementary Information

Supplementary Information 1.

## Data Availability

Bioinformatics scripts and DESeq2 results are available on GitHub (https://github.com/hayneslm/PAI-1_funcational_landscape.git). Raw sequencing results are available upon request.

## References

[1] Irving JA, Pike RN, Lesk AM, Whisstock JC (2000). Phylogeny of the serpin superfamily: Implications of patterns of amino acid conservation for structure and function. Genome Res..

[2] Huntington JA (2011). Serpin structure, function and dysfunction. J. Thromb. Haemost..

[3] Stein PE, Carrell RW (1995). What do dysfunctional serpins tell us about molecular mobility and disease?. Nat. Struct. Biol..

[4] Lawrence DA (1995). Serpin-protease complexes are trapped as stable acyl-enzyme intermediates. J. Biol. Chem..

[5] Silverman GA (2001). The serpins are an expanding superfamily of structurally similar but functionally diverse proteins. Evolution, mechanism of inhibition, novel functions, and a revised nomenclature. J. Biol. Chem..

[6] Huntington JA, Read RJ, Carrell RW (2000). Structure of a serpin-protease complex shows inhibition by deformation. Nature.

[7] Fredenburgh JC, Stafford AR, Weitz JI (2001). Conformational changes in thrombin when complexed by serpins. J. Biol. Chem..

[8] Peterson FC, Gettins PG (2001). Insight into the mechanism of serpin-proteinase inhibition from 2D [1H-15N] NMR studies of the 69 kDa alpha 1-proteinase inhibitor Pittsburgh-trypsin covalent complex. Biochemistry.

[9] Lawrence DA (2000). Partitioning of serpin-proteinase reactions between stable inhibition and substrate cleavage is regulated by the rate of serpin reactive center loop insertion into beta-sheet A. J. Biol. Chem..

[10] Khan MS (2011). Serpin inhibition mechanism: A delicate balance between native metastable state and polymerization. J. Amino Acids.

[11] Marijanovic EM (2019). Reactive centre loop dynamics and serpin specificity. Sci. Rep..

[12] Scott BM, Sheffield WP (2020). Engineering the serpin α(1)-antitrypsin: A diversity of goals and techniques. Protein Sci..

[13] Sprengers ED, Kluft C (1987). Plasminogen activator inhibitors. Blood.

[14] Alessi MC, Declerck PJ, De Mol M, Nelles L, Collen D (1988). Purification and characterization of natural and recombinant human plasminogen activator inhibitor-1 (PAI-1). Eur. J. Biochem..

[15] Vassalli JD, Sappino AP, Belin D (1991). The plasminogen activator/plasmin system. J. Clin. Invest..

[16] Cesarman-Maus G, Hajjar KA (2005). Molecular mechanisms of fibrinolysis. Br. J. Haematol..

[17] Dieval J, Nguyen G, Gross S, Delobel J, Kruithof EK (1991). A lifelong bleeding disorder associated with a deficiency of plasminogen activator inhibitor type 1. Blood.

[18] Lee MH, Vosburgh E, Anderson K, McDonagh J (1993). Deficiency of plasma plasminogen activator inhibitor 1 results in hyperfibrinolytic bleeding. Blood.

[19] Schleef RR, Higgins DL, Pillemer E, Levitt LJ (1989). Bleeding diathesis due to decreased functional activity of type 1 plasminogen activator inhibitor. J. Clin. Invest..

[20] Fay WP, Parker AC, Condrey LR, Shapiro AD (1997). Human plasminogen activator inhibitor-1 (PAI-1) deficiency: Characterization of a large kindred with a null mutation in the PAI-1 gene. Blood.

[21] Fay WP, Shapiro AD, Shih JL, Schleef RR, Ginsburg D (1992). Brief report: Complete deficiency of plasminogen-activator inhibitor type 1 due to a frame-shift mutation. N. Engl. J. Med..

[22] Carmeliet P (1993). Plasminogen activator inhibitor-1 gene-deficient mice. I. Generation by homologous recombination and characterization. J. Clin. Invest..

[23] Khan SS (2017). A null mutation in SERPINE1 protects against biological aging in humans. Sci. Adv..

[24] Morrow GB, Whyte CS, Mutch NJ (2021). A serpin with a finger in many PAIs: PAI-1's central function in thromboinflammation and cardiovascular disease. Front. Cardiovasc. Med..

[25] Fowler DM, Fields S (2014). Deep mutational scanning: A new style of protein science. Nat. Methods.

[26] Stefansson S (2004). Mutants of plasminogen activator inhibitor-1 designed to inhibit neutrophil elastase and cathepsin G are more effective in vivo than their endogenous inhibitors. J. Biol. Chem..

[27] Polderdijk SG (2017). Design and characterization of an APC-specific serpin for the treatment of hemophilia. Blood.

[28] Kretz CA, Tomberg K, Van Esbroeck A, Yee A, Ginsburg D (2018). High throughput protease profiling comprehensively defines active site specificity for thrombin and ADAMTS13. Sci. Rep..

[29] Kretz CA (2015). Massively parallel enzyme kinetics reveals the substrate recognition landscape of the metalloprotease ADAMTS13. Proc. Natl. Acad. Sci. USA.

[30] Stoop AA, Jespers L, Lasters I, Eldering E, Pannekoek H (2000). High-density mutagenesis by combined DNA shuffling and phage display to assign essential amino acid residues in protein-protein interactions: Application to study structure–function of plasminogen activation inhibitor 1 (PAI-I). J. Mol. Biol..

[31] Berkenpas MB, Lawrence DA, Ginsburg D (1995). Molecular evolution of plasminogen activator inhibitor-1 functional stability. EMBO J..

[32] Pannekoek H, van Meijer M, Schleef RR, Loskutoff DJ, Barbas CF (1993). Functional display of human plasminogen-activator inhibitor 1 (PAI-1) on phages: Novel perspectives for structure–function analysis by error-prone DNA synthesis. Gene.

[33] Love MI, Huber W, Anders S (2014). Moderated estimation of fold change and dispersion for RNA-seq data with DESeq2. Genome Biol..

[34] De Taeye B, Gils A, Declerck PJ (2004). The story of the serpin plasminogen activator inhibitor 1: Is there any need for another mutant?. Thromb. Haemost..

[35] Stoop AA, Eldering E, Dafforn TR, Read RJ, Pannekoek H (2001). Different structural requirements for plasminogen activator inhibitor 1 (PAI-1) during latency transition and proteinase inhibition as evidenced by phage-displayed hypermutated PAI-1 libraries. J. Mol. Biol..

[36] Chuang YJ, Swanson R, Raja SM, Bock SC, Olson ST (2001). The antithrombin P1 residue is important for target proteinase specificity but not for heparin activation of the serpin. Characterization of P1 antithrombin variants with altered proteinase specificity but normal heparin activation. Biochemistry.

[37] Owen MC, Brennan SO, Lewis JH, Carrell RW (1983). Mutation of antitrypsin to antithrombin. Alpha 1-antitrypsin Pittsburgh (358 Met leads to Arg), a fatal bleeding disorder. N. Engl. J. Med..

[38] Rashid Q, Kapil C, Singh P, Kumari V, Jairajpuri MA (2015). Understanding the specificity of serpin-protease complexes through interface analysis. J. Biomol. Struct. Dyn..

[39] Thomsen MC, Nielsen M (2012). Seq2Logo: A method for construction and visualization of amino acid binding motifs and sequence profiles including sequence weighting, pseudo counts and two-sided representation of amino acid enrichment and depletion. Nucleic Acids Res..

[40] Lawrence DA, Strandberg L, Ericson J, Ny T (1990). Structure–function studies of the SERPIN plasminogen activator inhibitor type 1. Analysis of chimeric strained loop mutants. J. Biol. Chem..

[41] Sherman PM (1992). Saturation mutagenesis of the plasminogen activator inhibitor-1 reactive center. J. Biol. Chem..

[42] Audenaert AM, Knockaert I, Collen D, Declerck PJ (1994). Conversion of plasminogen activator inhibitor-1 from inhibitor to substrate by point mutations in the reactive-site loop. J. Biol. Chem..

[43] Tucker HM, Mottonen J, Goldsmith EJ, Gerard RD (1995). Engineering of plasminogen activator inhibitor-1 to reduce the rate of latency transition. Nat. Struct. Biol..

[44] Gils A, Knockaert I, Declerck PJ (1996). Substrate behavior of plasminogen activator inhibitor-1 is not associated with a lack of insertion of the reactive site loop. Biochemistry.

[45] Lawrence DA, Olson ST, Palaniappan S, Ginsburg D (1994). Serpin reactive center loop mobility is required for inhibitor function but not for enzyme recognition. J. Biol. Chem..

[46] Mottonen J (1992). Structural basis of latency in plasminogen activator inhibitor-1. Nature.

[47] Knecht C, Krawczak M (2014). Molecular genetic epidemiology of human diseases: From patterns to predictions. Hum. Genet..

[48] Ng PC, Henikoff S (2001). Predicting deleterious amino acid substitutions. Genome Res..

[49] Adzhubei IA (2010). A method and server for predicting damaging missense mutations. Nat. Methods.

[50] Karczewski KJ (2020). The mutational constraint spectrum quantified from variation in 141,456 humans. Nature.

[51] Fowler DM, Stephany JJ, Fields S (2014). Measuring the activity of protein variants on a large scale using deep mutational scanning. Nat. Protoc..

[52] Stein A, Fowler DM, Hartmann-Petersen R, Lindorff-Larsen K (2019). Biophysical and mechanistic models for disease-causing protein variants. Trends. Biochem. Sci..

[53] Sherman PM (1995). Identification of tissue-type plasminogen activator-specific plasminogen activator inhibitor-1 mutants. Evidence that second sites of interaction contribute to target specificity. J. Biol. Chem..

[54] Fernandez-de-Cossio-Diaz J, Uguzzoni G, Pagnani A (2020). Unsupervised inference of protein fitness landscape from deep mutational scan. Mol. Biol. Evol..

[55] Levin EG, Santell L (1987). Conversion of the active to latent plasminogen activator inhibitor from human endothelial cells. Blood.

[56] Yee A (2021). Phage display broadly identifies inhibitor-reactive regions in von Willebrand factor. J. Thromb. Haemost..

[57] Yee A, Tan FL, Ginsburg D (2013). Functional display of platelet-binding VWF fragments on filamentous bacteriophage. PLoS One.

[58] Sim N-L (2012). SIFT web server: Predicting effects of amino acid substitutions on proteins. Nucleic Acids Res..

[59] Lin Z (2011). Structural Basis for recognition of urokinase-type plasminogen activator by plasminogen activator inhibitor-1*. J. Biol. Chem..

[60] Zeslawska E (2003). Crystals of urokinase type plasminogen activator complexes reveal the binding mode of peptidomimetic inhibitors. J. Mol. Biol..

[61] Stout TJ, Graham H, Buckley DI, Matthews DJ (2000). Structures of active and latent PAI-1: A possible stabilizing role for chloride ions. Biochemistry.

