# Supplementary material for: Deep mutational scanning of the plasminogen activator inhibitor-1 functional landscape
Source: Sci Rep. 2021 Sep 22;11:18827. doi: 10.1038/s41598-021-97871-7 (PMC8458277; doi:10.1038/s41598-021-97871-7)
Supplement: Supplementary file 1 — Supplementary Information 1. [file 41598_2021_97871_MOESM1_ESM.docx]

**SUPPLEMENTARY MATERIALS**

SI Table 1. Primer Sequences

| Primer | 5’ → 3’ Sequence |
| --- | --- |
| pAY-FE PAI-1 Cloning Primers | |
| pAY-FE PAI-1 For | GCCGGCGCGCCAGAACAAAAACTCATCTCAGAAGAGGATC  TGGGTGGAGGTTCAGTGCACCATCCCCCATCCTAC |
| pAY-FE PAI-1 Rev | ACCTGCGGCCGCTGAACCACCTCCGGGTTCTATCACTTGGCCCAT |
| pAY-FE PAI-1 Mutagenesis Primers | |
| PAI-1 Mutagenesis For | TTCTCGGCCTAGCCGGCCGG |
| PAI-1 Mutagenesis Rev | GTCCTTGTAGTCACCACCACCTGCGGCC |
| pAY-FE-VWF-A3 Cloning Primers | |
| pAY-FE-VWF-A3 For | GGCGCGCCATCGGGAGGAGGGTCCCCTGCACCTGACTGCAGCCAGCCCCTGGACGTGATC |
| pAY-FE-VWF-A3 Rev | ACCTGCGGCCGCACCGCCGCCGCACAGTTTGTGGAGGAAGGAATTGCCCAAGGT |
| Sanger Sequencing/Colony PCR Primers | |
| pAYE Sequencing For | TTCATGCTGCCGGCTTTCTCGG |
| pAYE Sequencing Rev | TCGTCATCGTCCTTGTAGTCACCACCA |
| HTS Sequencing Primers | |
| PAI-1 Amplicon 1 For | NNNNNNGAACAAAAACTCATCTCAGAAGAGGATCTG |
| PAI-1 Amplicon 1 Rev | NNNNNNGGGTGAGAAAACCACGTTGCG |
| PAI-1 Amplicon 2 For | NNNNNNTTTCAGCAGGTGGCGCAG |
| PAI-1 Amplicon 2 Rev | NNNNNNCTTGTCATCAATCTTGAATCCCATAGCTGC |
| PAI-1 Amplicon 3 For | NNNNNNATGCTCCAGCTGACAACAGGA |
| PAI-1 Amplicon 3 Rev | NNNNNNTGTGGTGCTGATCTCATCCTTGTT |
| PAI-1 Amplicon 4 For | NNNNNNGCCCTCCGGCATCTGTACAAG |
| PAI-1 Amplicon 4 Rev | NNNNNNTTGCTTGACCGTGCTCCGG |
| PAI-1 Amplicon 5 For | NNNNNNAAGCTGGTCCAGGGCTTCATG |
| PAI-1 Amplicon 5 Rev | NNNNNNCCCAAGCAAGTTGCTGATCATACC |
| PAI-1 Amplicon 6 For | NNNNNNATTCATCATCAATGACTGGGTGAAGAC |
| PAI-1 Amplicon 6 Rev | NNNNNNCTGGAGTCGGGGAAGGGAG |
| PAI-1 Amplicon 7 For | NNNNNNAATGCCCTCTACTTCAACGGC |
| PAI-1 Amplicon 7 Rev | NNNNNNGGGCGTGGTGAACTCAGTATAG |
| PAI-1 Amplicon 8 For | NNNNNNCCCATGATGGCTCAGACCAAC |
| PAI-1 Amplicon 8 Rev | NNNNNNGGCAGAGAGAGGCACCTCT |
| PAI-1 Amplicon 9 For | NNNNNNAGCATGTTCATTGCTGCCCC |
| PAI-1 Amplicon 9 Rev | NNNNNNTTCAGTCTCCAGGGAGAACTTGG |
| PAI-1 Amplicon 10 For | NNNNNNCAGGCTGCCCCGCCT |
| PAI-1 Amplicon 10 Rev | NNNNNNACGTGGAGAGGCTCTTGGTC |
| PAI-1 Amplicon 11 For | NNNNNNAGACAGTTTCAGGCTGACTTCAC |
| PAI-1 Amplicon 11 Rev | NNNNNNGGGGGCCATGCGGGC |
| PAI-1 Amplicon 12 For | NNNNNNTCATCCACAGCTGTCATA |
| PAI-1 Amplicon 12 Rev | NNNNNNTGCGGCCGCTGAACCACCTCC |

SI Table 2. PAI-1 amino acid residues spanned by HTS amplicons

| **PAI-1 Amplicon** | **Residues Sequenced** | **Residues Analyzed** |
| --- | --- | --- |
| 1 | V1 – D29 | V1-K28 |
| 2 | A26 – A60 | D29-Q56 |
| 3 | G52 – W86 | Q57-M83 |
| 4 | E81 – L116 | G84-F114 |
| 5 | P111 – K145 | R115-T144 |
| 6 | H143 – K176 | K145-W175 |
| 7 | Q174 – F208 | K176-F208 |
| 8 | K207 – E242 | N209-E243 |
| 9 | Y241 – L275 | K244-V274 |
| 10 | L273 – S310 | L275-L309 |
| 11 | S308 – S344 | S310-V343 |
| 12 | V343 – P379 | S344-P379 |

SI Table 3. Observed frequencies for χ^2^ test comparing experimental results to gnomAD database

|  | **Scored by phage display** | **Present in gnomAD** | **Not present in gnomAD** |
| --- | --- | --- | --- |
| **Functional** | 826 | 110 | 716 |
| **Non-functional** | 1137 | 92 | 1045 |
| **Total** | 1963 | 202 | 1761 |

**SI Fig. 1.**

**
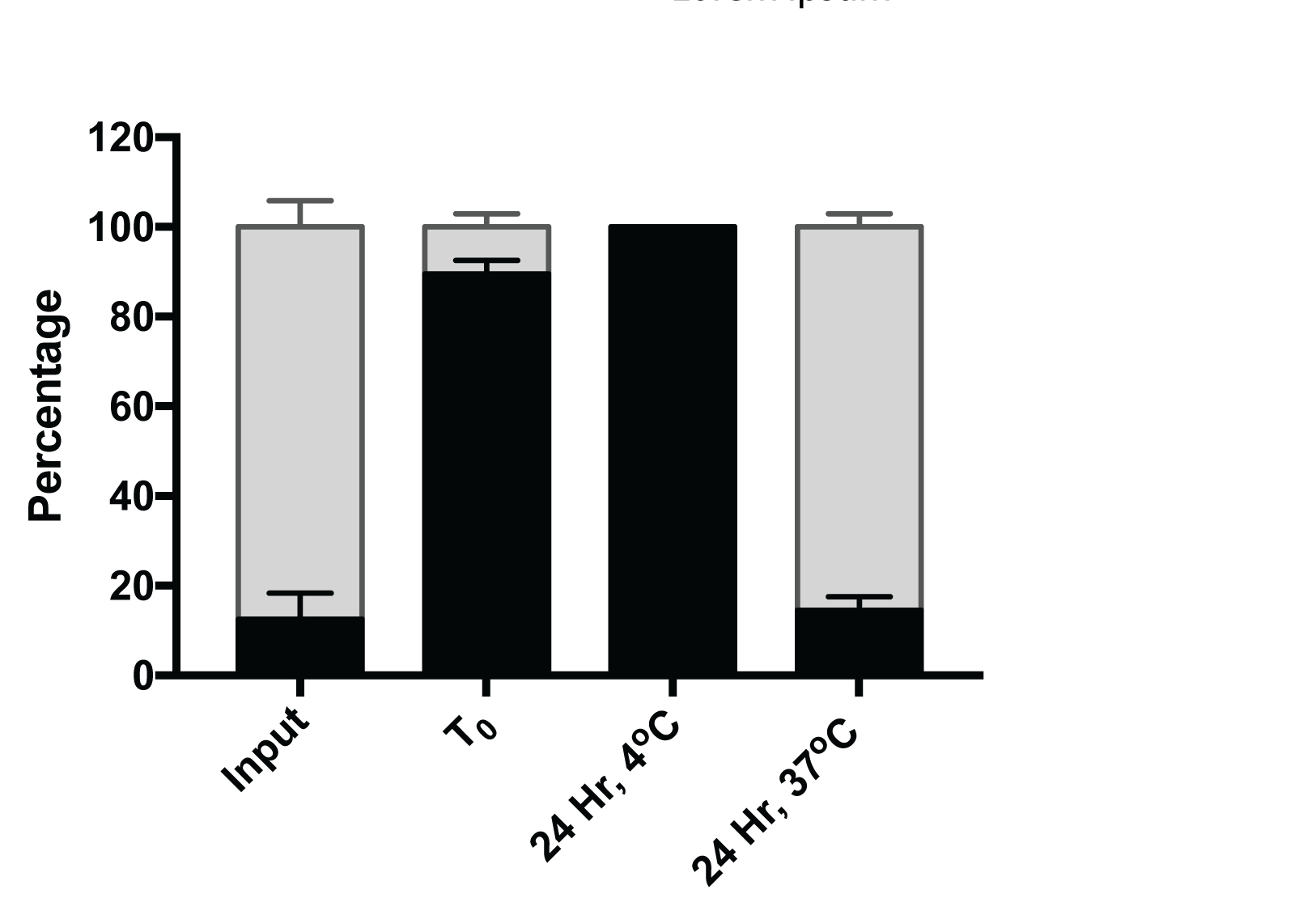
**

The percent active phage was determined at time zero (T_0_) or following incubation for 24 h at either 4^o^ C or 37^o^ C. Input phage contained ~15% phage-displayed PAI-1 and ~85% phage-displayed von Willebrand Factor A3 domain (Ser1681-Cys1872, a non-uPA reactive protein). At T_0_, ~90% of the phage selected for uPA binding were phage-displayedPAI-1. After 24 h incubation at 4^o^C, 100% of the selected phage were phage-displayed PAI-1 compared to ~10 % after 24 h at 37^o^C.
